# Zyflamend, a unique herbal blend, induces cell death and inhibits adipogenesis through the coordinated regulation of PKA and JNK

**DOI:** 10.1080/21623945.2020.1803642

**Published:** 2020-08-11

**Authors:** Dexter Puckett, Mohammed Alquraishi, Dina S. Alani, Samah Chahed, Victoria D. Frankel, Dallas Donohoe, Brynn Voy, Jay Whelan, Ahmed Bettaieb

**Affiliations:** a Department of Nutrition, University of Tennessee Knoxville, Knoxville, TN, USA; b Tennessee Agricultural Experiment Station, University of Tennessee Institute of Agriculture, Knoxville, TN, USA; c Graduate School of Genome Science and Technology, University of Tennessee, Knoxville, TN, USA; d Department of Biochemistry, Cellular and Molecular Biology, University of Tennessee, Knoxville, TN, USA

**Keywords:** Zyflamend, adipogenesis, lipolysis, AMPK, PKA, JNK

## Abstract

The prevalence of obesity and its comorbidities has sparked a worldwide concern to address rates of adipose tissue accrual. Recent studies have demonstrated a novel role of Zyflamend, a blend of natural herbal extracts, in regulating lipid metabolism in several cancer cell lines through the activation of the AMPK signalling pathway. Yet, the role of Zyflamend in adipogenic differentiation and lipid metabolism remains largely unexplored. The objective of this study is to investigate the effects of Zyflamend on white 3T3-MBX pre-adipocyte differentiation and elucidate the molecular mechanisms. We demonstrate that Zyflamend treatment altered cell cycle progression, attenuated proliferation, and increased cell death of 3T3-MBX pre-adipocytes. In addition, treatment with Zyflamend inhibited lipid accumulation during the differentiation of 3T3-MBX cells, consistent with decreased expression of lipogenic genes and increased lipolysis. Mechanistically, Zyflamend-induced alterations in adipogenesis were mediated, at least in part, through the activation of AMPK, PKA, and JNK. Inhibition of AMPK partially reversed Zyflamend-induced inhibition of differentiation, whereas the inhibition of either JNK or PKA fully restored adipocyte differentiation and decreased lipolysis. Taken together, the present study demonstrates that Zyflamend, as a novel anti-adipogenic bioactive mix, inhibits adipocyte differentiation through the activation of the PKA and JNK pathways.

**Abbreviation**: 7-AAD: 7-amino-actinomycin D; ACC: acetyl-CoA carboxylase; AKT: protein kinase B; AMPK: AMP-activated protein kinase; ATGL: adipose triglyceride lipase; C/EBPα: CCAAT-enhancer binding protein alpha; DMEM: Dulbecco’s Modified Eagle Medium; DMSO: dimethyl sulphoxide; DTT: dithiothreitol; EGTA: ethylene glycol-bis-(2-aminoethyl)-N,N,N’,N’-tetraacetic acid; ERK: extracellular signal–regulated kinases; FASN: fatty acid synthase; FBS: foetal bovine serum; GLUT: glucose transporter; HSL: hormone-sensitive lipase; IR: insulin receptor; IRS: insulin receptor substrate; JNK: c-JUN N-terminal kinase; MGL: monoacylglycerol lipase; NaF: sodium fluoride; NF-κB: nuclear factor kappa-light-chain-enhancer of activated B cells; PBS: phosphate buffered- saline; PCB: pyruvate carboxylase; PDE: phosphodiesterase; PKA: protein kinase cAMP-dependent; PMSF: phenylmethylsulfonyl fluoride; PPARγ: perilipin peroxisome proliferator-activated receptor gamma; PREF-1: pre-adipocyte factor 1; PVDF: polyvinylidene fluoride; RIPA: radio-immunoprecipitation assay; SDS-PAGE: sodium dodecyl sulphate polyacrylamide gel electrophoresis; SEM: standard error of the mean; SOX9: suppressor of cytokine signalling 9; TGs: triacylglycerols.

## Significance

We identified novel properties of Zyflamend as a potential anti-adipogenic, anti-lipogenic, and pro-lipolytic herbal mix. We firmly believe that these novel and unexpected findings have significant translational implications and will be of major interest to a wide spectrum of scientists. Additionally, while we believe that Zyflamend may have potential therapeutic implications for obesity and its complications, we do not suggest the use of this combination as primary treatment for obesity. However, there is a possibility that it could be used either as an adjuvant with medically-approved standard therapies or in a preventative manner.

## Introduction

Obesity and its comorbidities have emerged as an epidemic of substantial proportions [1,2]. Obesity affects 36.5% of all Americans [2] and often shows a direct correlation to excessive caloric intake [2,3]. The burden of excess adipose tissue on metabolic homoeostasis can be drastic and remains a major risk factor for the development of numerous diseases [2]. Moreover, obesity has been associated with the impaired functioning of critical organs including the kidney, liver, pancreas, and heart [1,4–6]. As a result, there is a high demand for innovative and effective approaches targeting adipose tissue and obesity in both, a therapeutic and preventative manner.

Adipogenesis, the process in which an immature pre-adipocyte differentiates and matures leading to increased lipid accumulation, is an important component in the development of obesity. Once mature, these cells act as energy storage sites and can modulate processes essential to the homoeostasis and function of a variety of other tissues. Disruption of this process offers an alluring target in both the prevention and treatment of obesity. Thus, targeting varying aspects of adipogenesis has been at the forefront of obesity research. Although current anti-obesity medications promoting fat loss do exist, the safety of these medications is often disputed [7,8] and dedication to new prevention and treatment strategies must be continued. A growing body of evidence suggests that phytochemicals and natural herbal extracts, when used at pharmacological doses, could be capable of delaying the onset and progression of obesity through a variety of mechanisms including differentiation, cell death, and lipolysis.

Zyflamend, a unique blend of ten herbal extracts, was proven to have anti-inflammatory and anti-tumorigenic properties at physiologically relevant doses [9]. However, Zyflamend’s biological roles beyond cancer metabolism have been relatively unexplored. Recently, we demonstrated that Zyflamend activates AMP-activated protein kinase (AMPK) *in vitro* across various cell lines [10] and *in vivo* in a preclinical experimental model of U.S. diet-induced metabolic disorder [11]. Given the critical role of AMPK in regulating adipogenesis, glucose uptake, fatty acid metabolism, and mitochondrial function, it is likely that Zyflamend may also influence body mass and glycaemic control. Notably, we recently reported that Zyflamend supplementation for 4 weeks significantly reduced adiposity, improved insulin sensitivity, and reduced plasma levels of non-essential fatty acids [11]. These changes were concomitant with increased AMPK phosphorylation and inhibition of acetyl CoA-carboxylase (ACC) in adipose tissue [11], suggesting that Zyflamend may exhibit anti-adipogenic and/or pro-lipolytic properties. In this study, we examined the *in vitro* effects of Zyflamend on the differentiation of white adipocytes and investigated the potential molecular mechanisms mediating its actions.

## Material and methods

### Chemicals and reagents

Trypsin, media, and sera utilized in cell culture experiments were purchased and acquired from Gibco (Thermo Fisher Scientific, Waltham, MA). Zyflamend (Table S1) was purchased from New Chapter Inc. (Brattleboro, VT). Antibodies, both primary and secondary, were obtained from numerous sources (Table 1). Chemical reagents including digitonin, protease inhibitors cocktail, dithiothreitol (DTT), propidium iodide, percoll, phenylmethylsulfonyl fluoride (PMSF), sodium fluoride (NaF), sodium deoxycholate, ethylene glycol-bis-(2-aminoethyl)-N,N,N’,N’-tetraacetic acid (EGTA), Triton X-100, PKA inhibitor (H89), and JNK inhibitor (SP600125) were obtained from Millipore-Sigma (Burlington, MA). AMPK inhibitor (BML275) was purchased from Santa Cruz Biotechnology (Santa Cruz, CA). General caspases inhibitor (Z-VAD.fmk) was obtained from Calbiochem (La Jolla, CA).

**Table 1.** List of primary antibodies and conditions of use

Antibodies	Source	Host	Dilution
ACC	Cell Signalling Technology	Rabbit	1:2,000
Adiponectin	Abcam	Rabbit	1: 1,000
AMPK	Cell Signalling Technology	Rabbit	1:5,000
Cleaved caspase-3 (C.Casp.3)	Cell Signalling Technology	Rabbit	1:5,000
C/EBP	Santa Cruz Biotechnology	Mouse	1:1,000
c-FOS	Santa Cruz Biotechnology	Mouse	1:1,000
c-JUN	Santa Cruz Biotechnology	Mouse	1:1,000
CPT1a	Abcam	Mouse	1: 1,000
Cyclin-D1	Cell Signalling Technology	Rabbit	1:5,000
Cyclin-D2	Cell Signalling Technology	Rabbit	1:2,500
Cyclin-D3	Cell Signalling Technology	Rabbit	1:5,000
FASN	Santa Cruz Biotechnology	Mouse	1:5,000
Fibronectin	Santa Cruz Biotechnology	Mouse	1: 1,000
GLUT4	Santa Cruz Biotechnology	Mouse	1:5,000
HSL	Cell Signalling Technology	Rabbit	1:1,000
JNK1/2	Santa Cruz Biotechnology	Mouse	1:1,000
P38	Santa Cruz Biotechnology	Mouse	1:1,000
PCB	Santa Cruz Biotechnology	Mouse	1:5,000
Perilipin	Santa Cruz Biotechnology	Mouse	1:1,000
PKA-RIIβ	Santa Cruz Biotechnology	Mouse	1:1,000
Phospho-ACC^S9^	Cell Signalling Technology	Rabbit	1:1,000
Phospho-AMPK^T172^	Cell Signalling Technology	Rabbit	1:2,500
Phospho-c-JUN^S63^	Santa Cruz Biotechnology	Mouse	1:1,000
Phospho-HSL^S563^	Cell Signalling Technology	Rabbit	1:1,000
Phospho-HSL^S565^	Cell Signalling Technology	Rabbit	1:1,000
Phospho-HSL^S660^	Cell Signalling Technology	Rabbit	1:1,000
Phospho-JNK1/2^T183/Y185^	Santa Cruz Biotechnology	Mouse	1:1,000
Phospho-P38^T180/Y182^	Santa Cruz Biotechnology	Mouse	1:1,000
Phospho-PKA Substrate (RRX^S*^/^T*^)	Cell Signalling Technology	Rabbit	1:5,000
PPARγ	Cell Signalling Technology	Rabbit	1:1,000
PREF-1	Cell Signalling Technology	Rabbit	1:1,000
SOX9	Santa Cruz Biotechnology	Mouse	1:1,000
α5 Integrin	Santa Cruz Biotechnology	Mouse	1: 1,000
β-Actin	Santa Cruz Biotechnology	Mouse	1:20,000

### Cell culture

3T3-L1 MBX (ATCC ® CRL-3242) white pre-adipocytes were purchased from ATCC. Brown precursor and 3T3-L1 MBX cells were cultured in DMEM containing 25 mM glucose, 10% FBS, 50 U/ml penicillin, and 50 μg/ml streptomycin. To induce cell differentiation, pre-adipocytes were grown to 100% confluency in culture medium containing 10% FBS, then switched to differentiation media containing 10% FBS, 20 nM insulin and 1 nM triiodothyronine [T3] for 48 hr Adipocyte differentiation was induced by switching to a fresh differentiation media supplemented with 5 µM dexamethasone, 0.5 mM isobutylmethylxanthine, and 0.125 mM indomethacin for 48 hr (induction media). Cells were then returned to differentiation medium until they exhibited a fully differentiated phenotype characterized by the accumulation of fat droplets.

### Zyflamend treatment

Zyflamend was dissolved in DMSO at a concentration of 800 mg/ml for the stock solution. Cells cultured in 60 mm dishes were treated with Zyflamend for the indicated duration and at the indicated concentration. Treatments were stopped with two washes with ice-cold phosphate-buffer saline (PBS). Finally, plates were flash frozen in liquid nitrogen and stored at −80ºC until further analyses.

### Cytotoxicity assay

Cytotoxicity assays were performed using sulforhodamine B (Millipore-Sigma) as previously described [12] with modification. Briefly, 3T3-MBX pre-adipocytes were treated with Zyflamend at 37°C in an atmosphere of 10% CO_2_ for the indicated time. After treatment, cells were fixed with 17% trichloroacetic acid in PBS, and cellular proteins were stained for 10 min at room temperature with 0.4% sulforhodamine B in 1% acetic acid solution. The plates were then washed with water, dried, and the stain was dissolved in 10 mM Tris (pH 9). Quantification of sulforhodamine B stain was carried out using the Synergy™ HTX Multi-Mode microplate reader (BioTek Instruments, Inc. Winooski, VT) at a wavelength of 540 nm. The relative survival rates of cells were determined by dividing the absorbance observed for a given treatment by the absorbance detected in the control cells treated with DMSO and expressed as a fold change. In some experiments, the MTT (3-[4,5-dimethylthiazol-2-yl]-2,5-diphenyltetrazolium bromide) cytotoxicity assay was performed as previously described with modification [13]. Briefly, pre- (20,000 cells/well) or fully differentiated 3T3-L1-MBX adipocytes (5,000 cells/well) were plated in a 96-well plate for 24 hr, then starved in 200 μl DMEM culture medium containing 0.1% FBS for 12 hr prior to an additional 24 hr treatment with Zyflamend (200 μg/ml). At the end of the experiment, 40 μl of the MTT solution (5 mg/ml) was added to each well for 4 hr, then cell culture was removed and the dye was dissolved in 100 μl 10% SDS solution overnight at 37°C. Relative cytotoxicity was determined by measuring the absorbance at 570 nm using the Synergy™ HTX Multi-Mode microplate reader.

### Quantification of lipid accumulation and oil red o staining

Quantification of lipid accumulation was performed as previously described [14]. Briefly, on day 12 of differentiation, cells were fixed with 10% PBS buffered formalin for at least 12 hr at 4°C. Cells were stained for one hour with filtered Oil Red O solution (5 g/L in isopropyl alcohol), washed with distilled water, and examined using the Leica DMI8 inverted microscope (Leica Microsystems Inc. Buffalo Grove, IL).

### Caspase activity

Following Zyflamend treatment, caspase-3 activity was determined as previously described with modification [15]. Briefly, 2 × 10^6^ cells were lysed in 50 μl cell lysis buffer (50 mM HEPES, pH 7.4, 100 mM NaCl, 0.1% CHAPS, 1 mM DTT, 0.1 mM EDTA) in a 96 well plate and incubated at −20°C for 30 min. Next, 50 μl of the caspases assay buffer solution (50 mM HEPES, pH 7.4, 100 mM NaCl, 0.1% CHAPS, 10 mM DTT, 0.1 mM EDTA and 10% glycerol) containing 50 µM of the caspase-3 substrate (Ac-DEVD-pNA; Calbiochem, La Jolla, CA) was added to each well and incubated at 37°C for 30 min. Caspase-3 activity was determined by measuring the absorbance at 405 nm using the Synergy™ HTX Multi-Mode microplate reader and was presented as caspase-3 activity relative to control cells.

### Western blotting analysis

Cells were lysed in radio-immunoprecipitation assay (RIPA: 10 mM Tris-HCl, pH 7.4, 150 mM NaCl, 0.1% sodium dodecyl sulphate [SDS], 1% Triton X-100, 1% sodium deoxycholate, 5 mM EDTA, 1 mM NaF, 1 mM sodium orthovanadate and protease inhibitors) buffer as we previously described [16]. Clarification of lysates occurred through centrifugation at 15,000 g for 10 min, and the level of protein concentration was quantified using the bicinchoninic acid assay kit (Thermo Scientific™ Pierce™ BCA Protein Assay, Thermo Fisher Scientific, Waltham, MA). Proteins (10–30 μg) were resolved by SDS-PAGE and then transferred on polyvinylidene fluoride (PVDF) membrane. Immunoblotting of lysates was performed with primary antibodies (Table 1), and following incubation with secondary antibodies, proteins were detected using Luminata™ Western Chemiluminescent HRP Substrate (Millipore-Sigma). Immunoreactive bands were quantified based on pixel intensity using FluorChem Q Imaging software (Alpha Innotech Corp, San Leandro, CA). Data is represented as being either normalized to the respective unphosphorylated form or to β-actin as loading controls for each respective condition.

### RNA extraction and real-time PCR

RNA was extracted from cells using TRIzol Plus RNA Isolation System (Invitrogen, Carlsbad, CA). The concentration of RNAs was determined using the NanoDrop® ND-1000 spectrophotometer (Thermo Scientific). Reverse transcription was performed on equal RNA samples using the High-Capacity cDNA Synthesis Kit (Applied Biosystems, Austin, TX). Expression of different genes was assessed by quantitative real-time PCR using SsoAdvanced™ Universal SYBR® Green Supermix (Bio-Rad Laboratories, Inc., Hercules, CA) and the Bio-Rad CFX96™ system. Primer sets used in these experiments are described in Table 2. The relative abundance of target gene mRNA was measured using the ∆∆CT method and normalized to 18S ribosomal RNA.

**Table 2.** List of primers used to quantitate the mRNA levels of *markers of differentiation.*

*Gene*	*Forward 5ʹ->3’*	*Reverse 5ʹ->3’*
*18s*	*GCAATTATTCCCCATGAACG*	*GGCCTCACTAAACCATCCAA*
*Adioq*	*TGTTCCTCTTAATCCTGCCCA*	*CCAACCTGCACAAGTTCCCTT*
*Cebpa*	*GGTCAACAGGAGAATCTCCCAG*	*CTCTGTTTTATGCTGTTATGGGTGA*
*Dlk1*	*CCCAGGTGAGCTTCGAGTG*	*GGAGAGGGGTACTCTTGTTGAG*
*Fabp4*	*GCTTTGCCACAAGGAAAGTG*	*CATAACACATTCCACCACCA*
*Fasn*	*AGAGATCCCGAGACGCTTCT*	*GCCTGGTAGGCATTCTGTAGT*
*Retn*	*CTGTCCAGTCTATCCTTGCACAC*	*CAGAAGGCACAGCAGTCTTGA*

### Annexin V staining

Annexin V staining was performed as previously described [17] with modification. Briefly, 3T3-MBX pre-adipocytes were exposed to Zyflamend for the indicated duration, then washed with PBS and resuspended in 0.5 ml of PBS containing 10% FBS. Equal volumes of the Guava Nexin reagents were added, and cells were incubated for 10 min at room temperature in the dark. Cells (5,000) were then analysed using the Guava® easyCyte ^TM^ Flow Cytometer (Millipore-Sigma). Intensities of fluorescence emitted by annexin V-fluorescein isothiocyanate (FITC) and 7-amino-actinomycin D (7-AAD) were collected on PM1 and PM2 channels, respectively. Both viable (negative for both annexin V-FITC and 7-AAD) and apoptotic cells (early apoptosis: positive for annexin V-FITC, but negative for 7-AAD, and late apoptosis positive for both: annexin V-FITC and 7-AAD) were quantified using the InCyte™ and Guava Suite Software Package (Luminex Corp., Austin, TX).

### Cell cycle analysis

Cell cycle analysis was performed in observation of the cellular DNA content and level of propidium iodide stain as previously described [18] with minor alterations. Briefly, 3T3-MBX pre-adipocytes were treated with Zyflamend for the designated duration, then washed with PBS and fixed in 70% ethanol for 12 hr at 4°C. Cells were then washed twice with cold PBS and incubated in RNase solution (100 U/ml) in PBS for 15 min at 37°C. Cells were then incubated in propidium iodide solution (10 μg/ml in PBS) overnight at 4°C. A total number of 5000 cells was acquired using the Guava® easyCyte ^TM^ Flow Cytometer. Cells that are labelled with propidium iodide are then plotted as a function of cells at each intensity level (Y axis). Fluorescence intensity of propidium iodide (relative to DNA content and shown as the X-axis on the representative histogram) was quantified using the InCyte™ and Guava Suite Software package.

### 2-deoxyglucose uptake assay

2-deoxyglucose uptake was determined using the colorimetric glucose uptake assay kit (Biovison Inc., Milpitas, CA). Briefly, fully differentiated 3T3-MBX adipocytes were starved overnight in low glucose (1 mM) and 0.1% FBS media, then treated with insulin for 30 min in the presence of 2-deoxyglucose. Cells were then washed with ice-cold PBS, and the accumulated 2-deoxyglucose was quantitated using the Synergy™ HTX Multi-Mode microplate reader.

### Statistical analysis

Data analysis relied on the JMP Pro 13.2 program (SAS Institute, Chapel Hill, NC) and was represented as means + standard error of the mean (SEM). For all statistical analyses, unpaired heteroscedastic two-tail Student’s t-test was used, and differences were considered highly significant at p < 0.01 and significant at p < 0.05. Single symbol (such as *) corresponds to p < 0.05, while double symbols (such as **) corresponds to p < 0.01.

## Results

### Zyflamend inhibits adipocyte differentiation in a dose dependent manner

**Figure 1.**
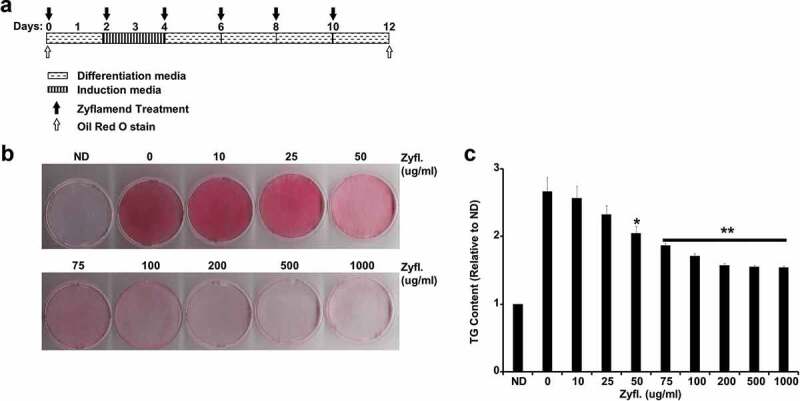
Dose Dependent Effect of Zyflamend on 3T3-MBX adipocyte differentiation. (a) An overview of the experimental design and the differentiation procedure. 3T3-MBX were differentiated in the presence of increasing doses of Zyflamend for 12 days. Cells were fixed and stained with Oil Red O. (b) Oil Red O staining of non-differentiated (ND; Day 1 of differentiation), fully differentiated adipocytes (0 μg/ml; day 12 of differentiation), and cells treated with the indicated concentrations of Zyflamend for 12 days. (c) Oil Red O stain was extracted, and its absorbance (520 nm) was quantitated. Bar graph represents data from six independent experiments, and are expressed as mean + SEM. *p < 0.05, **p < 0.01 indicate significant difference between non-treated and Zyflamend-treated cells

We previously reported that Zyflamend is a polyherbal supplement composed of 10 botanical extracts (Supplementary Table S1), significantly reduced adiposity and activated AMPK signalling [11]. Based on these *in vivo* effects and the established role of AMPK in regulating lipid metabolism and adipogenesis, we tested the effects of Zyflamend on multiple aspects of adipocyte differentiation *in vitro*. 3T3-MBX white pre-adipocytes were induced to differentiate into fully mature adipocytes and simultaneously treated with increasing concentrations of Zyflamend as outlined in (Figure 1(a)). Cells were then stained using the fat-specific dye Oil Red O to monitor the effects of treatment with increasing concentrations of Zyflamend on lipid accumulation and differentiation. Adipocytes treated with Zyflamend showed a dose-dependent decrease in lipid accumulation (Figure 1(b)). Quantification of lipid accumulation with Oil Red O staining indicated a significant reduction in cellular triglyceride content with Zyflamend doses as low as 50 μg/ml in comparison to vehicle (DMSO) controls (Figure 1(c)). Notably, cells treated with 1 mg/ml of Zyflamend exhibited blunted differentiation on day 12 and were comparable to the non-differentiated cells on day 1.

**Figure 2.**
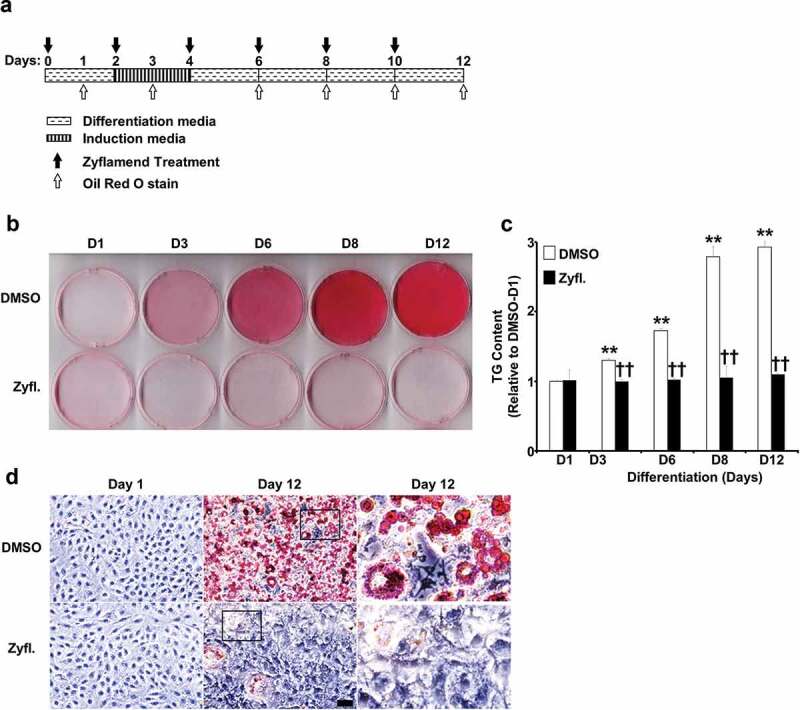
A physiologically relevant dose of Zyflamend inhibits 3T3-MBX adipocyte differentiation. (a) An overview of the experimental design and the differentiation procedure. (b) 3T3-MBX pre-adipocytes were treated with DMSO or 200 μg/ml of Zyflamend and differentiated, as described in the methods section. Freshly prepared solutions of DMSO and Zyflamend were added at each change of media. At different days of differentiation, cells were fixed and stained with Oil Red O, then the dye was extracted, and its absorbance (520 nm) quantitated (c). Bar graph represents data from six independent experiments, and are expressed as mean + SEM. *p < 0.05, **p < 0.01 indicate significant difference between the indicated time and day one of differentiation. †*p* < 0.05, ††*p* < 0.01 indicate significant difference between Zyflamend and control (DMSO) treated cells. (d) Phase contrast images of non-differentiated (Day 1), fully differentiated adipocytes (Day 12), and cells treated with Zyflamend (200 μg/ml) for 12 days. Scale bar: 100 μm. Images in the far right panels are a zoomed capture of the boxed areas on Day 12 of differentiation

To sequentially monitor the effect of Zyflamend on lipid accumulation, cells were induced to differentiate in the presence or absence of Zyflamend (200 μg/ml) and stained with Oil Red O at 1, 3, 6, 8 and 12 days post-induction (Figure 2(a)). This dose was chosen as a physiologically relevant level representative of the maximum plasma concentration of 2 μM of its constituent curcumin that was reported in humans upon supplementation [19]. At this human-equivalent dose, Zyflamend significantly attenuated adipogenesis and accumulation of lipid droplets (Figure 2(b-c)). Additionally, microscopic analysis revealed a drastic difference between control and Zyflamend treated cells. As shown in Figure 2(d), control cells accumulated fat droplets and exhibited a fully differentiated phenotype on day 12 of differentiation. However, only a small percentage of cells accumulated fat droplets in the Zyflamend-treated group, confirming the anti-adipogenic effects of this herbal blend (Figure 2(d)). However, the precise mechanism and stage in which Zyflamend repressed differentiation were yet to be determined.

### A physiologically relevant dose of Zyflamend causes cell cycle arrest and induces apoptosis in 3T3-MBX pre-adipocytes

Adipocyte differentiation is a complex process that requires the coordinated actions of several hormonal and nutritional factors, and the integration of multiple signalling pathways [20–23]. Based on findings from cancer studies, Zyflamend-induced alteration in adipogenesis can be caused by a plethora of signalling pathways, including, but not limited to those involved in the regulation of cell cycle, proliferation and/or death, lipolysis, lipogenesis, contact inhibition, clonal expansion, and/or trans-differentiation into brown-like adipocytes. Thus, we conducted a detailed analysis of the effects of Zyflamend on pre-adipocyte proliferation, cell cycle, cell death, and differentiation.

**Figure 3.**
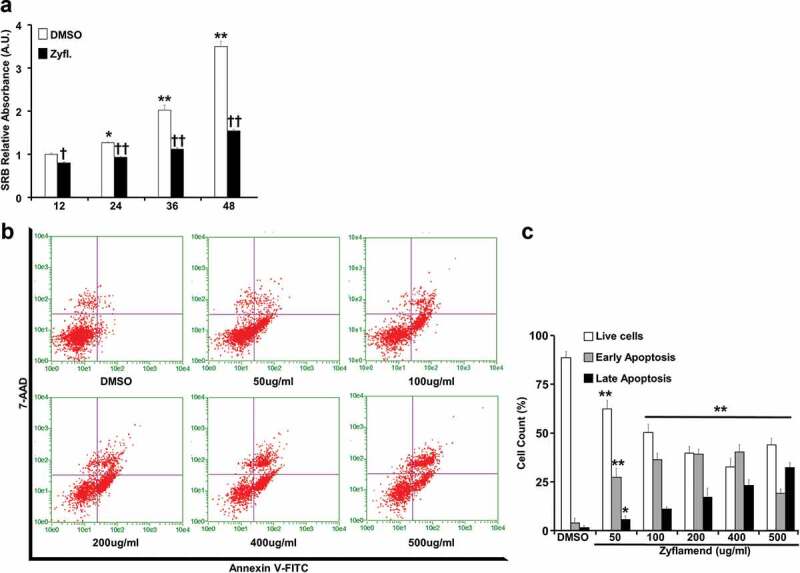
A physiologically relevant dose of Zyflamend causes cell cycle arrest and induces apoptosis in 3T3-MBX pre-adipocytes. (a) 3T3-MBX pre-adipocytes were treated with DMSO or 200 μg/ml of Zyflamend for the indicated time. Zyflamend toxicity and cell proliferation were assessed using the SRB cytotoxicity assay as detailed in the method section. Bar graphs represent the intensity of SRB staining reflective of the cell number and presented as means + SEM. *p < 0.05, **p < 0.01 indicate significant difference between cell survival rate at the indicated time point and 12 hr. †*p* < 0.05, ††*p* < 0.01 indicate significant difference between non-treated and Zyflamend-treated cells. (b) 50% confluent cells were treated with increasing concentrations of Zyflamend, and then labelled with Annexin V-FITC and 7-AAD. Representative dot plots are shown. Annexin V positive and 7-AAD negative cells (lower right quadrants) represent early stages of apoptosis. Whereas cells that are positive for both annexin V and 7-AAD (upper right quadrants) are in the late stages of apoptosis. (**c)** Bar graphs represent live, early, and late apoptotic cells are presented as means + SEM of at least three independent experiments. *p < 0.05, **p < 0.01 indicate significant difference between the indicated concentration and control cells treated with the vehicle DMSO

First, we used the sulforhodamine B cytotoxicity assay, also used as an indirect measure of cell proliferation [24], to examine whether Zyflamend exhibits cytotoxic effects on 3T3-MBX pre-adipocytes, and whether it alters cell proliferation. Proliferating pre-adipocytes were cultured in the presence of Zyflamend (200 μg/ml) or DMSO (0.25 μl/ml) as vehicle control for up to 48 hr. Consistent with cancer studies [25–27], Zyflamend significantly reduced cell proliferation (Figure 3(a)). Next, we examined changes in the cell cycle in proliferating 3T3-MBX pre-adipocytes in response to Zyflamend treatment. Accordingly, cells at 50% confluency were treated with increasing concentrations of Zyflamend, and changes in cell cycle progression were examined. Analysis of cell distribution among the different phases of the cell cycle according to their DNA contents revealed that Zyflamend caused a dose-dependent cell cycle arrest at G2/M phase. The percentages of cells in the G2/M cell cycle phase were significantly higher in Zyflamend-treated cells and increased from 1.3 ± 0.7% in DMSO-treated control cells to 17.1 ± 3.4%, 23.4 ± 4.1%, and 32.6 ± 5.7% in cells exposed to 200, 400, or 500 μM of Zyflamend for 24 hr, respectively (Supplementary Figure S1A-B). Additionally, we observed remarkable changes in response to Zyflamend treatment in the expression of D-type cyclins (Supplementary Figure S1C-D) which play a critical role in coordinating cell proliferation, survival, and differentiation, and are the core of cell cycle machinery. Indeed, the expression of cyclins D1 and D2 was significantly attenuated in Zyflamend treated cells compared to controls, whereas, the expression of cyclin D3 was high on day 1 of differentiation then decreased gradually during differentiation (Supplementary Figure S1C-D). Next, the levels of early and late apoptosis in 3T3-MBX pre-adipocytes treated with DMSO or Zyflamend were measured using annexin V- FITC and 7-AAD as markers of early and late cellular apoptosis, respectively (Figure 3(b-c)). Zyflamend-treated pre-adipocytes exhibited a dose-dependent increase in both early and late apoptosis as judged by the elevated number of cells that stained positive for annexin V (x-axis), and 7-AAD (y-axis) (Figure 3(c)). As the dose of Zyflamend increased, so did the percentages of cells in late apoptosis (Figure 3(c)). Taken together, our data show that Zyflamend elicited strong effects towards the suppression of pre-adipocyte growth and proliferation, while simultaneously inducing cell death.

### Patterns of differentiation correlate with the expression of adipogenic markers

**Figure 4.**
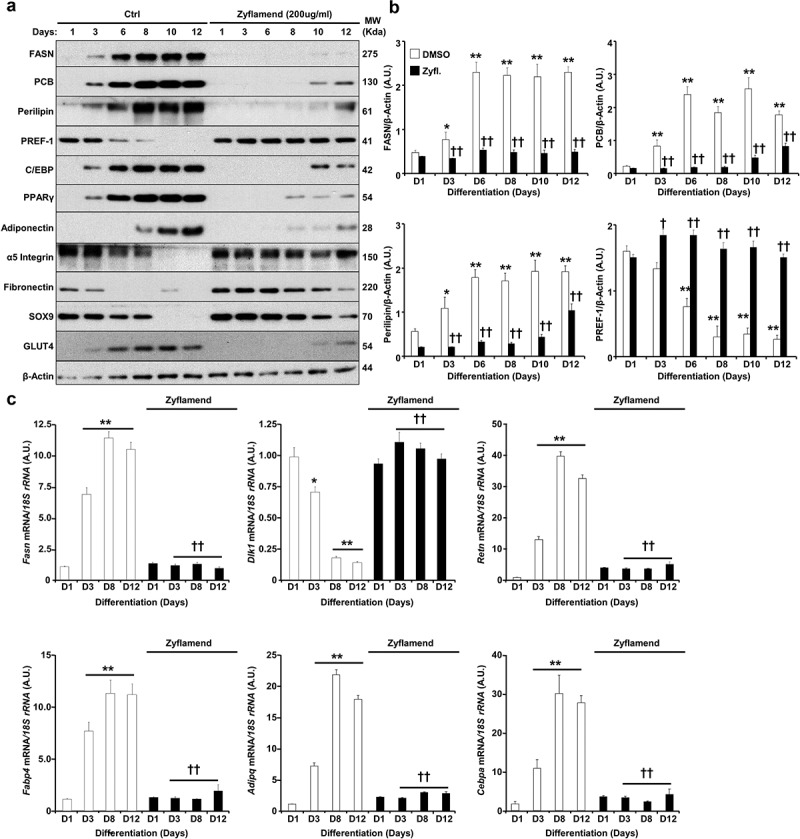
Zyflamend alters the expression of adipogenic markers during the differentiation of 3T3-MBX adipocytes. (a) Immunoblots of adipogenic markers in 3T3-MBX cells treated or non-treated with Zyflamend (200 μg/ml) at varying stages of differentiation. Lysates were blotted for β-actin to control for loading. Representative immunoblots from three independent experiments are shown. (b) Bar graphs represent the indicated protein normalized to β-Actin as means + SEM. *p < 0.05, **p < 0.01 indicate significant difference between the indicated time points and day 1 for each cell type. †*p* < 0.05, ††*p* < 0.01 indicate significant difference between Zyflamend and control (DMSO) treated cells. **(c**) Quantitative (q)RT-PCR of *Fasn, Dlk1, Retn, Fabp4, Adipq*, and *Cebpa* mRNA levels in control and Zyflamend-treated (200 μg/ml) cells at various days of differentiation. Data are normalized to *18S ribosomal RNA* (*18S rRNA*). Results are representative of three independent experiments and data are expressed as mean + SEM. *p < 0.05, **p < 0.01 indicate significant difference between indicated time points and day 1 for each treatment. †*p* < 0.05, ††*p* < 0.01 indicate significant difference between Zyflamend and control (DMSO) treated cells

To further investigate the effects of Zyflamend on adipogenesis, we determined the expression levels of several key adipogenic markers; fatty acid synthase (FASN), pyruvate carboxylase (PCB), perilipin, peroxisome proliferator-activated receptor gamma (PPARγ), CCAAT-enhancer binding protein alpha (C/EBPα), adiponectin, and glucose transporter 4 (GLUT4). Consistent with previous reports [28,29], FASN, PCB, C/EBP, adiponectin, PPARγ exhibited a progressive increase in expression during adipocyte differentiation in control cells treated with the vehicle DMSO (Figure 4(a)). However, cells treated with Zyflamend (200 μg/ml) failed to exhibit increased expression of these proteins that characterize the progression of adipocyte differentiation (Figure 4(a-b)), with only modest expression of some proteins detectable in the late stages of differentiation. In contrast, the expression of pre-adipocyte factor 1 (PREF-1), a marker of pre-adipocytes, persisted at comparable levels through day 12 in Zyflamend-treated cells, compared to the expected decline as observed in control cells. Levels of other proteins associated with the non-differentiated state of white adipocytes, such as α5 integrin, fibronectin, and suppressor of cytokine signalling 9 (SOX9), also declined with the progression of adipogenesis in control cells, but persisted longer with Zyflamend treatment (Figure 4(a)). In addition, we examined changes in mRNA levels of genes associated with the non-differentiated state of white adipocytes, such as *Delta like non-canonical notch ligand 1*(*Dlk1*; a.k.a. *pre-adipocyte factor 1*), and genes involved in the differentiation of white adipocytes, including fatty acid synthase (*Fasn*), resistin (*Retn), Fatty acid-binding protein* (a.k.a. *Adipocyte protein P2; Fabp4*/*Ap2), adiponectin* (*Adipoq), and* CCAAT/enhancer-binding protein alpha (*c/ebp1a*). As shown in Figure 4(c), while control cells exhibited a marked increase in *Fas*, resistin, *c/ebp1a*, and *adiponectin*, the expression of these genes was significantly abolished in Zyflamend-treated cells. In contrast, *pref1* mRNA levels are significantly reduced in differentiated adipocytes compared to pre-adipocytes, but remained higher in Zyflamend-treated cells compared to controls (Figure 4(c)), further confirming our Western blotting data and the anti-adipogenic effects of Zyflamend.

To further assess the effects of Zyflamend on differentiation, and confirm that cells treated with Zyflamend remain undifferentiated, we evaluated basal and insulin-stimulated 2-deoxy-glucose uptake. In agreement with the reduced expression of Glut4 in response to Zyflamend (Figure 4(a)), cells treated with Zyflamend (200 μg/ml) exhibited a robust and significant decrease in insulin-stimulated glucose uptake compared with control cells treated with the vehicle DMSO (Supplementary Figure S2). Collectively, these findings demonstrate that Zyflamend attenuates differentiation of white adipocytes, as established by Oil Red O staining, lipid accumulation, protein and mRNA expression of differentiation markers, and glucose uptake.

### Zyflamend dose-dependently regulates lipogenic and lipolytic proteins in mature adipocytes

**Figure 5.**
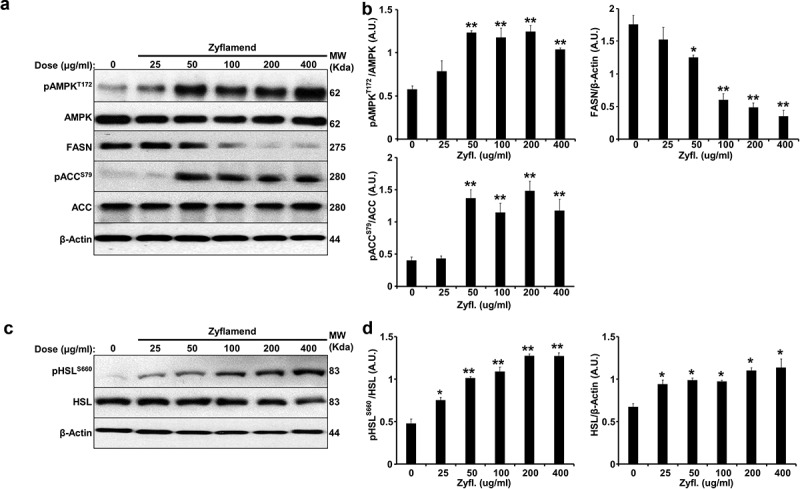
Zyflamend inhibits lipogenesis and induces lipolysis in fully differentiated 3T3-MBX adipocytes. Total lysates from fully differentiated 3T3-MBX adipocytes treated with the indicated concentrations of Zyflamend for 24 hr were immunoblotted for markers of lipogenesis (a-b) and lipolysis (c-d). Representative immunoblots from three independent experiments are shown. (b-d) Bar graphs represent pAMPK^T172^/AMPK, FASN/β-Actin, pACC^S79^/ACC, pHSL^S660^/HSL, and HSL/β-Actin as means + SEM. *p < 0.05, **p < 0.01 indicate significant difference between the indicated concentration of Zyflamend and control untreated cells

In an effort to determine the effects of Zyflamend on mature adipocytes, we treated fully differentiated 3T3-MBX cells with Zyflamend and assessed the effects on markers of de novo lipogenesis and lipolysis. Additionally, we examined whether Zyflamend could alter AMPK phosphorylation [30] at threonine 172 (T172), a phosphorylation site that is required for AMPK activation and is associated with lipolysis and inhibition of lipogenesis in adipose tissue and adipocytes [31]. Zyflamend treatment induced the phosphorylation and activation of AMPK in a dose dependent manner (Figure 5(a-b)). Acetyl-CoA carboxylase (ACC) is a rate-limiting enzyme in de novo lipogenesis, and phosphorylation by AMPK on serine 79 inhibits ACC activity. Zyflamend dose-dependently increased phosphorylation of ACC, in parallel with activation of AMPK (Figure 5(a-b)) [32]. In addition, Zyflamend significantly decreased the abundance of fatty acid synthase (FAS), which, along with ACC, regulates the synthesis of fatty acids from glucose (Figure 5(a-b)).

We evaluated changes in the activity of hormone-sensitive lipase (HSL), a rate-limiting enzyme that regulates lipolysis in human and rodent adipose tissue, to determine if Zyflamend also promotes lipolysis in mature adipocytes. HSL is phosphorylated at multiple sites, where each modification regulates both the translocation and activity of the enzyme. Protein kinase A (PKA) phosphorylates HSL at serine site 660 (S660) which promotes HSL translocation to lipid droplets and triacylglycerol hydrolysis [33]. Treatment of fully-differentiated 3T3-MBX adipocytes with Zyflamend resulted in a dose dependent increase in the phosphorylation of HSL at S660 (Figure 5(c-d)), suggesting enhanced lipolysis.

### Zyflamend inhibits the maturation of adipocytes through the coordinated regulation of PKA and JNK

**Figure 6.**
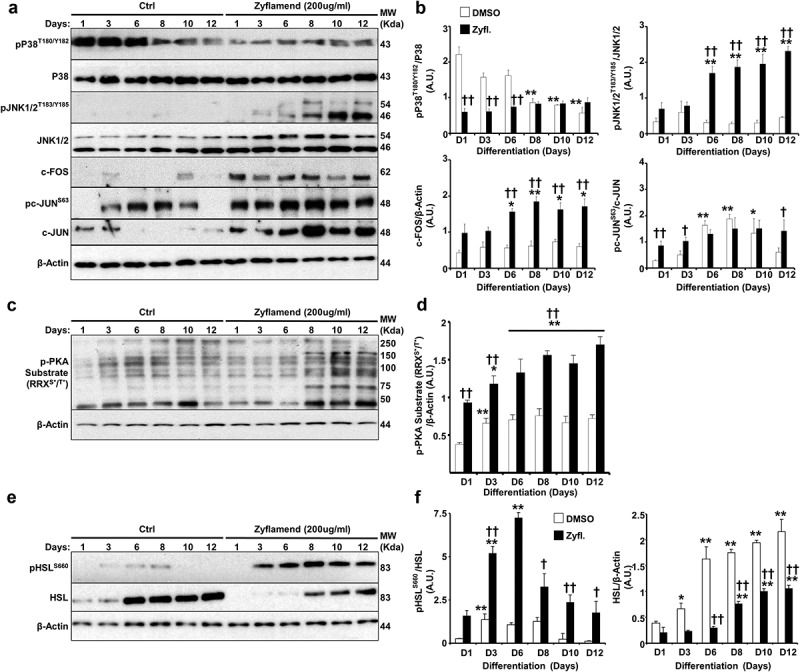
Zyflamend activates PKA signalling pathway in differentiating 3T3-MBX adipocytes. (a-b) Total cell lysates from control and Zyflamend treated cells for the entire duration of differentiation were immunoblotted for phosphorylated P38, JNK, c-JUN, their respective unphosphorylated proteins, c-FOS, and β-Actin to control for loading. (a) Representative immunoblots from three independent experiments are shown. (b) Bar graphs represent pP38^T180/Y182^/P38, pJNK^T183/Y185^/JNK, pc-JUN^S63^/c-JUN, and c-FOS/β-Actin as means + SEM. p < 0.05, **p < 0.01 indicate significant difference between indicated time points and day 1 for each treatment. †*p* < 0.05, ††*p* < 0.01 indicate significant difference between Zyflamend and control (DMSO) treated cells. (c) Immunoblots of phosphorylated PKA substrate in 3T3-MBX cells treated or non-treated with Zyflamend (200 μg/ml) for the entire duration of differentiation. Representative immunoblots from three independent experiments are shown. (d) Bar graphs represent phosphorylated PKA substrate/β-Actin as means + SEM. *p < 0.05, **p < 0.01 indicate significant difference between indicated time points and day 1 for each treatment. †*p* < 0.05, ††*p* < 0.01 indicate significant difference between Zyflamend and control (DMSO) treated cells. (e) Immunoblots of pHSL^S660^, and HSL in 3T3-MBX cells treated or non-treated with Zyflamend (200 μg/ml) for the entire duration of differentiation. Representative Immunoblots from three independent experiments are shown. (f) Bar graphs represent pHSL^S660^/HSL, and HSL/β-Actin as means + SEM. *p < 0.05, **p < 0.01 indicate significant difference between the indicated time points and day 1 for each treatment. †*p* < 0.05, ††*p* < 0.01 indicate significant difference between Zyflamend and control (DMSO) treated cells

**Figure 7.**
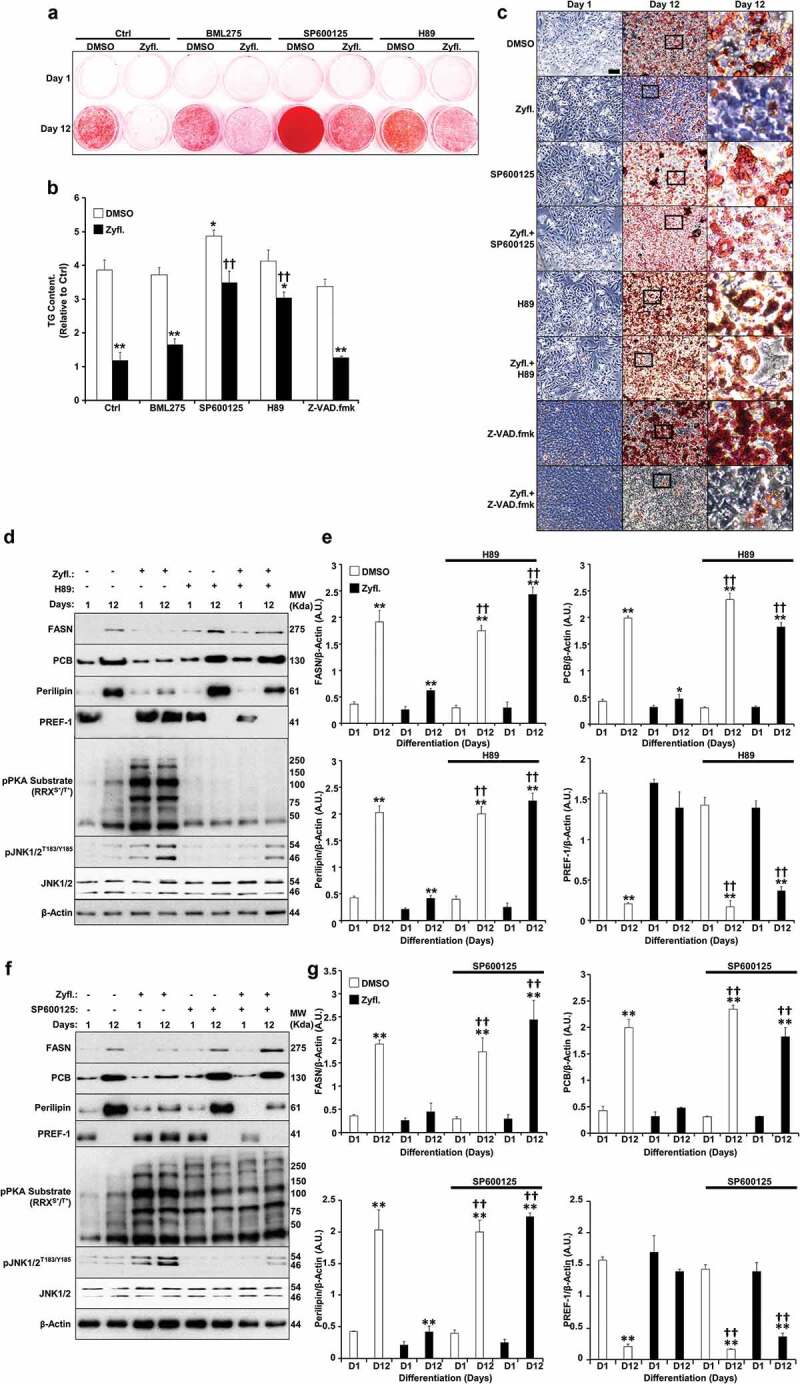
Inhibition of PKA and JNK abrogates the effects of Zyflamend on differentiation (a-b) Oil Red O-stained images of non-differentiated (Day 1) and differentiated adipocytes (Day 12) treated or non-treated with AMPK (BML275), JNK (SP600125), PKA (H89) or apoptosis (Z-VAD.fmk) inhibitors for the entire duration of differentiation. (b) Oil Red O stain was extracted, and its absorbance (520 nm) was quantitated. Bar graph represents data from at least six independent experiments, and data are expressed as mean + SEM. *p < 0.05, **p < 0.01 indicate significant difference between non-treated and Zyflamend-treated cells. †*p* < 0.05, ††*p* < 0.01 indicate significant difference between cells treated with the indicated inhibitor together with Zyflamend and cells treated with Zyflamend only. (c) Phase contrast images of non-differentiated (Day 1) and differentiated adipocytes (Day 12) treated or non-treated with Zyflamend, SP600125, H89, or Z-VAD.fmk (alone or in combination with Zyflamend). Images in the far right panels are a zoomed capture of the boxed areas on Day 12 of differentiation. Scale bar: 100 μm. (d-g) Immunoblots of adipogenic markers in 3T3-MBX cells treated or non-treated with Zyflamend (200 μg/ml) with or without PKA inhibitor (H89; d-e) or JNK inhibitor (SP600125; f-g). Lysates were blotted for β-actin to control for loading. Representative Immunoblots from three independent experiments are shown. e-g) Bar graphs represent the indicated protein normalized to β-Actin as means + SEM. *p < 0.05, **p < 0.01 indicate significant difference between day 1 and day 12 of differentiation for each treatment. †*p* < 0.05, ††*p* < 0.01 indicate significant difference between inhibitor-treated and non-treated cells

A number of studies have shown the requirement of crosstalk between cAMP-PKA and MAP kinase signalling pathways in orchestrating adipocyte differentiation and functions [34]. While the role of Zyflamend in activating PKA has not been previously addressed, a number of studies have shown that Zyflamend activates both c-jun N-terminal kinase (JNK) and the MAP kinase P38 in various cell types [9,35]. Thus, to begin to determine the mechanism underlying the anti-adipogenic and pro-lipolytic effects of Zyflamend, and the possible involvement of MAP kinases in mediating these effects, we examined the activation of PKA and MAP kinases during the course of differentiation of 3T3-MBX cells. Our data show that Zyflamend (200 μg/ml) had no effects on changes in extracellular signal–regulated kinases 1/2 (ERK1/2) and protein kinase B (AKT) phosphorylation during differentiation (data not shown). However, a marked decreased in P38 phosphorylation, concomitant with a significant increase in JNK phosphorylation, particularly on day 6 of differentiation, was observed in Zyflamend-treated cells compared to controls (Figure 6(a-b)). Zyflamend also increased the phosphorylation and activation of the JNK canonical downstream effectors, namely c-FOS and c-JUN (Figure 6(a-b)). Furthermore, Zyflamend treatment led to a higher PKA activity across the differentiation process, in comparison to the control cells as judged by the phosphorylation of PKA substrates (Figure 6(c-d)). In addition, PKA-mediated phosphorylation of HSL at S660 was significantly higher in Zyflamend-treated cells compared to controls (Figure 6(e-f)). Collectively, our results suggest that PKA, AMPK, P38, and/or JNK signalling pathways are mechanisms that potentially mediate the block in adipocyte differentiation induced by Zyflamend. Therefore, we investigated whether inhibition of AMPK, PKA, P38, or JNK could abolish the effects of Zyflamend on 3T3-MBX differentiation. BML275 (a.k.a. compound C) was also used to inhibit AMPK specifically. As shown in Figure 7(a-c), inhibition of JNK or PKA with SP600125 and H89, respectively, lessened the effect of Zyflamend on differentiation as judged by Oil Red O staining and triglyceride levels. However, the inhibition of P38 using SB203580 did not rescue the effects of Zyflamend on differentiation (data not shown). On the other hand, inhibition of AMPK using BML275 only resulted in a moderate rescue of Zyflamend-induced attenuation of adipocyte differentiation and accumulation of lipid droplets (Figure 7(a-b)).

It has been proposed that the activation of PKA or JNK during adipocyte differentiation can lead to lipolysis and abolish adipogenesis [36,37]. However, little is known about the crosstalk between the two proteins in coordinating the hydrolysis of triglycerides and how this affects the overall differentiation process. To address this question, we used specific inhibitors for PKA (H89) and JNK (SP600125) and examined changes in PKA and JNK activity and phosphorylation in response to the combined treatment with Zyflamend and SP600125, or Zyflamend and H89, respectively. We also evaluated the effects of Zyflamend and H89 on the overall differentiation process by assessing changes in the protein expression of several adipocyte differentiation markers; FASN, PCB, Perilipin, and PREF-1. Inhibition of PKA markedly attenuated the phosphorylation and activation of JNK while rescuing the effects of Zyflamend on the expression of the differentiation markers (Figure 7(d-e)). On the other hand, while inhibition of JNK restored the differentiation of 3T3-MBX cells, Zyflamend-induced PKA activation remained unchanged (figure 7(f-g)). These data suggest that Zyflamend-induced activation of both JNK and PKA attenuates 3T3-MBX differentiation through lipolytic induction.

Next, because treatment with Zyflamend induced apoptosis in pre-adipocytes (Figure 3), we sought to investigate whether inhibition of apoptosis using carbobenzoxy-valyl-alanyl-aspartyl-[O-methyl]-fluoromethylketone (Z-VAD.fmk) could reverse the anti-adipogenic effects of Zyflamend. Z-VAD.fmk has been previously shown to relieve the anti-adipogenic effects of various stressors and restore pre-adipocytes differentiation [38–40]. Thus, we co-treated pre-adipocytes with Zyflamend and Z-VAD.fmk throughout the differentiation process and monitored adipocyte differentiation using Oil Red O. As shown in Figure 7(b-c), although treatment with the pan-caspase inhibitor Z-VAD.fmk was able to reduce Zyflamend-induced cell toxicity and caspase-3 activation (Supplementary Figure S3A-B), it failed to alleviate the anti-adipogenic effects of Zyflamend, suggesting that apoptosis is not the main molecular mechanism that mediates the anti-adipogenic effects of Zyflamend. Conversely, inhibition of JNK and PKA using SP600125 and H89, respectively, attenuated Zyflamend-induced cytotoxicity and caspase-3 activity (Fig. S3A-B) and partially restored adipocyte differentiation (Supplementary Figure S3A-B). Furthermore, both the MTT and caspase 3 activity assays revealed that the pro-apoptotic and cytotoxic effects of Zyflamend were significantly lower in fully differentiated adipocytes compared to pre-adipocytes (Supplementary Figure S3A-B). Additionally, Z-VAD.fmk-mediated inhibition of cell death did not prevent Zyflamend-induced phosphorylation of HSL at S660 on day 8 of differentiation (Supplementary Figure S3C).

## Discussion

Zyflamend is a polyherbal mix, whose efficacy as an anti-inflammatory and anti-cancer agent has been well characterized in a number of studies [41–43]. Interest in its use as an adjuvant with medically-approved standard cancer therapies or in a preventative manner gained traction due to its ability to modulate energy metabolism in cancer cells and its effectiveness at relatively low levels due to the synergistic effects of its components [19]. Nevertheless, its effects on metabolism and other homoeostatic functions in non-cancer cells remain largely unexplored. We recently reported that Zyflamend supplementation to C57Bl6/J mice fed a western diet resulted in a significant reduction in fat mass, but had no effects on overall body weight or growth rates [11]. Effects on adiposity were accompanied by significant changes in tissue purine metabolism, concomitant with increased AMPK phosphorylation in epididymal white adipose tissue. Accordingly, we sought to investigate the basis for these effects by analysing the effects of Zyflamend treatment on pre-adipocyte proliferation, differentiation, and metabolism *in vitro.*

This study is the first to explore the potential effects of Zyflamend treatment on white adipocyte differentiation and lipid accumulation and to elucidate the molecular mechanisms. In this study, we report the anti-adipogenic properties of Zyflamend, which attenuated pre-adipocyte proliferation and increased cell death. In addition, treatment with Zyflamend inhibited lipid accumulation during the differentiation of 3T3-MBX cells, consistent with a decreased expression of PPARγ and rate-limiting genes in de novo lipogenesis. These effects were mediated, at least in part, through the upregulation of AMPK, PKA and JNK activities as inhibition of AMPK partially reversed Zyflamend-induced inhibition of differentiation, while the inhibition of either JNK or PKA fully restored adipocyte differentiation and decreased lipolysis. Taken together, these findings identify Zyflamend as a novel regulator of adipogenesis, and suggest that Zyflamend supplementation could prove valuable for the prevention of obesity and its complications.

Adipocyte differentiation is a complex process that requires the integration of multiple signals and is regulated by several hormonal and nutritional factors [20–23]. Synchronous cell cycle re-entry and mitotic clonal expansion of committed pre-adipocytes are among the first steps leading to adipogenesis (Schema 1). However, previous research has been inconclusive on whether this is essential for optimal differentiation [44–46]. Studies using experimental models of obesity have further underscored the coordinated functions of cell cycle regulators in adipogenesis and whole body metabolic homoeostasis [47,48]. Alterations in cell cycle re-entry and mitotic clonal expansion may result in a notable impact on adipogenic differentiation. Here within, we demonstrate that Zyflamend treatment attenuated the proliferation of 3T3 MBX pre-adipocytes. Additionally, treatment with Zyflamend resulted in a dose dependent increase in both early and late apoptosis in pre-adipocytes. Furthermore, Zyflamend treatment interfered with the cell cycle machinery by reducing the levels of cyclins D1 and D2 and causing a G2/M cell cycle arrest in pre-adipocytes. In support of our findings, numerous phytochemicals have been shown to act as cell cycle regulators through altering cyclin protein expression [49]. Individual components of Zyflamend, such as curcumin [50] and rosemary [51], were both shown to alter the progression of the cell cycle. Pre-adipocytes treated with rosemary extracts exhibited reduced expression of cyclin-D1 and other cell cycle regulators such as cyclin-dependent kinase 4 and cyclin-dependent kinase inhibitor 1A (p21, Cip1) [52]. However, the use of single dietary bioactive compounds for both treatment and prevention of obesity has resulted in a lack of clinical success. Conversely, the combination of several phytochemicals and the subsequent effects of targeting multiple signal transduction pathways simultaneously through additive or synergistic actions has been proposed to be a better approach to combat obesity [49,53,54].

At the molecular level, Zyflamend abolished the differentiation of white adipocytes, at least in large part, through up-regulating PKA and JNK activities. Here, we report inhibition of PKA eliminated the effects of Zyflamend on differentiation and resulted in fully differentiated adipocytes in comparison to cells treated with Zyflamend alone. It is worth noting that the PKA’s precise contribution to adipogenesis remains controversial and warrants additional investigation. PKA has four regulatory (RIα, RIIα, RIβ, RIIβ) and four catalytic (Cα, Cβ, Cγ, Prkx) subunit isoforms that are differentially expressed and regulated between different mammalian tissues and species. Previous reports have shown that RIIβ is the primary PKA regulatory subunit in the adipose tissue of both, humans and rodents [55] and that mice lacking RIIβ exhibited resistance to high fat diet-induced obesity and associated metabolic alterations [55,56]. However, recent studies have demonstrated that the disruption of the ubiquitously expressed PKA-RIIα subunit in mice is also protective against diet-induced obesity, glucose intolerance, and hepatic steatosis [57]. Conversely, deletion of the *Prkarcb* gene, which encodes the catalytic subunit Cβ in mice exhibited increased fat mass and decreased fat-free mass compared with controls when fed a regular chow diet. However, when animals were fed a high fat diet, PKA-Cβ deficiency protected male, but not female, mice against diet-induced obesity [58]. Additionally, recent studies have shown that both the expression of PKA-RIIβ and stimulated PKA activity are differentially regulated between different fat depots in obese versus lean humans [59], further adding to the complexity of the role of PKA in adipogenesis and obesity. Here, we report that inhibition of PKA using H89 alleviated the anti-adipogenic effects of Zyflamend and restored lipid accumulation and the expression of adipogenic genes. These findings are in line with previous reports demonstrating that H89 enhances adipocyte differentiation [60]. Notably, treatment of 3T3-L1 cells with epidermal growth factor (EGF) activated PKA and blocked adipogenesis. Additionally, in a study by Li and colleagues, stimulation of PKA activity was suggested to inhibit the differentiation of 3T3-L1 pre-adipocytes, possibly through the modulation of the insulin signalling pathway and the down regulation of the phosphorylation of insulin receptor substrate 1 (IRS-1) [37]. In the same study, the authors demonstrated that, while forskolin-mediated activation of PKA inhibited adipogenesis in a dose-dependent manner, inhibition of PKA activity using H89 abolished the anti-adipogenic effects of forskolin [37]. Furthermore, activation of PKA by vinpocetine, a phosphodiesterase (PDE) type-1 inhibitor that increases cAMP and cGMP levels, resulted in the suppression of adipogenesis and lipid accumulation in 3T3-L1 cells [61]. These findings are consistent with the study by Li and colleagues showing that, reducing cAMP levels using a specific adenylate cyclase inhibitor (SQ22536; which would prevent PKA activation) promoted adipogenesis [37]. Nevertheless, and despite the controversies around PKA’s role in adipogenesis, it is well established that activation of PKA via phosphorylation is a critical step for the phosphorylation of perilipin and HSL, both of which are highly expressed in adipose tissue. The phosphorylation and activation of HSL trigger the recruitment of HSL to lipid droplets, where it mediates the hydrolysis of stored lipids in concert with adipose triglyceride lipase (ATGL) [62]. The combined action of the neutral lipases ATGL, HSL, and monoacylglycerol lipase (MGL), is essential for the hydrolysis of TAGs and the release of glycerol and NEFAs [63]. HSL can be phosphorylated and activated by PKA in response to catabolic hormone induction of the cAMP signalling cascade [63]. Other protein kinases including AMPK, ERK, glycogen synthase kinase-4, and Ca^2+^/calmodulin-dependent kinase II have also been shown to regulate HSL enzymatic activity through phosphorylation [63]. The regulation of HSL activation by phosphorylation is a critical and reversible process. Five critical phosphorylation sites on serine residues have been revealed including S563, S659, and S660 that are phosphorylated by PKA and the basal site (S565) that is phosphorylated by AMPK and Ca^2+^/calmodulin-dependent kinase II. Phosphorylation of HSL at the latter site prevents its activation by PKA [64]. Here, we report increased phosphorylation of HSL at the PKA regulatory site S660 by Zyflamend. However, the mechanism as to how Zyflamend activates the PKA/HSL signalling cascade is unknown, although many of its constituents have been shown to independently regulate PKA and HSL activities in several cell types including differentiating adipocytes [65–69].

Similar to PKA research, studies looking at the role of JNK in adipogenesis are inconclusive and produced mixed results with some studies supporting the anti-adipogenic and pro-lipolysis functions of JNK, while other reports arguing for a pro-adipogenic role. In a study by Kusuyama and colleagues, a differential role of the different JNK isoforms in regulating adipogenesis has been proposed [70]. The overexpression of the p46, but not p54 isoform increased adipogenesis while the inhibition of both isoforms suppressed adipogenesis [70]. Conversely, shRNA mediated knockdown of JNK1 and/or JNK2 had no impact on 3T3-L1 pre-adipocyte differentiation but drastically accelerated basal lipolysis [71]. Furthermore, the inhibition of JNK using phloretin, a glucose transporter (GLUT) inhibitor, induced adipogenesis in marrow stromal cells [36]. In addition, treatment with the JNK inhibitor SP600125 significantly increased lipid accumulation in ST2 cells [36]. Other studies, however, argue that JNK is not directly involved in the differentiation process of adipocytes [72]. While the role of JNK in adipogenesis is yet to be resolved, our study demonstrates an anti-adipogenic role of JNK in 3T3-MBX cells because SP600125-mediated inhibition of JNK fully rescued the effects of Zyflamend on adipogenesis. It is noteworthy to point out that inhibition of JNK activity reduces the phosphorylation of IRS1 on serine 307, a phosphorylation site that disrupts the interaction between the insulin receptor (IR) and IRS1 [73] which alter the transduction of insulin signalling and glucose uptake in white adipocytes [74]. JNK also inhibits insulin receptor substrate 2 (IRS2) through a similar mechanism [75]. Both IRS-1 and IRS-2 play a critical role in adipocyte differentiation through regulating glucose uptake as well as the expression of adipogenic proteins C/EBPα and PPARγ [76]. Altering the function of IRS-1 and IRS-2 was reported to impair the adipogenesis and differentiation processes of both white [77] and brown [78] adipocytes, supporting the anti-adipogenic function of JNK. However, additional studies are necessary to determine the precise mechanisms underlying its role in adipocyte differentiation. In addition, future research aimed at elucidating the crosstalk mechanisms between PKA and JNK in halting adipogenesis and promoting lipolysis is warranted.

In conclusion, we report the anti-adipogenic properties of Zyflamend, which caused a G2/M cell cycle arrest in white 3T3-MBX pre-adipocytes, attenuated pre-adipocyte proliferation, and increased cell death. In addition, treatment with Zyflamend inhibited lipid accumulation during the differentiation of 3T3-MBX cells, with a concomitant decrease in the expression of lipogenic genes and increased lipolysis (Schema 1). These effects were mediated, at least in part, through the upregulation of AMPK, PKA, and JNK activities. Inhibition of either JNK or PKA fully restored adipocyte differentiation and decreased lipolysis (Schema 1). However, AMPK inhibition using compound C partially rescued adipogenesis. Taken together, these findings identify Zyflamend as a novel regulator of pre-adipocytes survival and adipogenesis, and suggest that Zyflamend supplementation could prove valuable for the prevention of obesity and its complications. However, it is important to note that many of the potentially desirable effects may have been mediated by Zyflamend’s cytotoxic effects, and future studies targeting its efficacy are warranted.

**Schematic 1:**
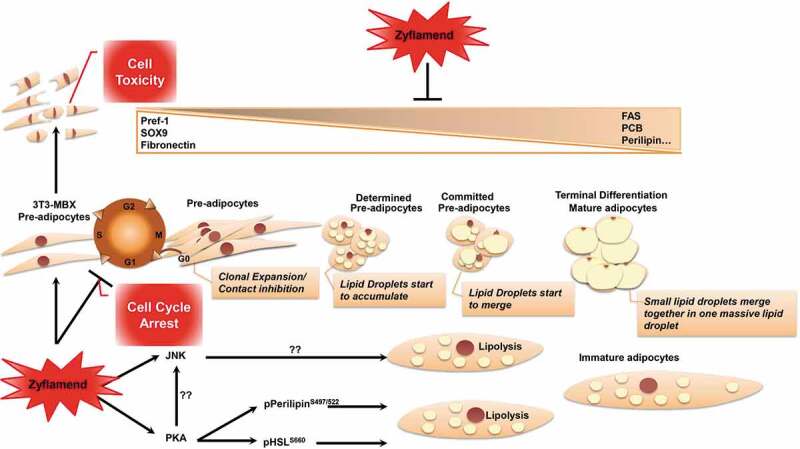
Overview of Zyflamend’s effects on adipocyte differentiation, lipolysis, and adipogenesis

A basic overview of how pre-adipocytes mature and differentiate is included as a reference. Zyflamend induced cell cycle arrest and exhibited cytotoxic effects on 3T3-MBX pre-adipocytes. Additionally, Zyflamend prevented the decrease in the levels of PREF-1, SOX9, and fibronectin (proteins associated with the pre-adipocyte state of 3T3-MBX cells) and reduced the level of FASN, PCB, perilipin, C/EBP, adiponectin, and PPARγ (proteins that promote the differentiation and formation of fully mature adipocytes). Furthermore, Zyflamend increased the activation of PKA, accompanied by a subsequent increase in perilipin and HSL, resulting in enhanced lipolysis. Finally, Zyflamend activation of PKA led to increased activation of JNK.

## Supplementary Material

Supplemental Material
