# Supplementary material for: Zyflamend, a unique herbal blend, induces cell death and inhibits adipogenesis through the coordinated regulation of PKA and JNK
Source: Adipocyte. 2020 Aug 11;9(1):454–71. doi: 10.1080/21623945.2020.1803642 (PMC7469463; doi:10.1080/21623945.2020.1803642)
Supplement: Supplemental Material [file KADI_A_1803642_SM0903.zip › suppl caption and table.docx]

**Supplementary Figure 1. A physiologically relevant dose of Zyflamend induces cell cycle arrest and alters the expression of the D type cyclin proteins.** **A**) Cell cycle analysis and assessment of DNA content in 3T3-MBX pre-adipocytes treated with DMSO or the indicated concentration of Zyflamend. Representative histogram distributions for each treatment are shown with the DNA content (Propidium iodide fluorescence intensity) on the X-axis and cell count on Y-axis **B**). Bar graphs represent the percentages of cells in each phase of the cell cycle, which were estimated using the Guava Suite Software package and are presented as means + SEM from three independent experiments. **p*<0.05, ***p*<0.01 indicate significant difference between the indicated concentration of Zyflamend and control cells treated with the vehicle DMSO. **C**) Immunoblots of cell cycle regulators cyclins D1, D2, and D3 in cells treated with 200 μg/ml of Zyflamend at various stages of differentiation. Representative immunoblots from three independent experiments are shown. **D)** Bar graphs represent cyclins D1, D2 and D3 normalized to β-Actin as a loading control and presented as means + SEM. **p*<0.05, ***p*<0.01 indicate significant difference between the indicated time point and day 1 of differentiation. †*p*<0.05, ††*p*<0.01 indicate significant difference between Zyflamend and control (DMSO-treated) cells.

**Supplementary Figure 2. Zyflamend inhibits glucose uptake.** 3T3-MBX pre-adipocytes were treated with DMSO or 200 μg/ml of Zyflamend and differentiated as described in the methods section. On day 12, cells were starved overnight in low glucose (1mM) and 0.1% FBS media, then treated with insulin for 30 min in the presence of 2-deoxyglucose. 2-deoxyglucose uptake was quantified and presented as means + SEM of three independent experiments. Graph represents data from four independent experiments, and data are expressed as mean + SEM. **p*<0.05, ***p*<0.01 indicate significant difference between the insulin-treated and non-treated cells. †*p*<0.05, ††*p*<0.01 indicate significant difference between Zyflamend and control (DMSO-treated) cells.

**Supplementary Figure 3. Inhibition of PKA and JNK alleviates Zyflamend-induced cellular toxicity and apoptosis. A**) 3T3-MBX pre-adipocytes and mature adipocytes (Day 12 of differentiation) were treated with freshly prepared solutions of DMSO or Zyflamend (200 μg/ml) for 24 hr Zyflamend toxicity was assessed using the MTT cytotoxicity assay as detailed in the method section. Bar graphs represent the intensity of formazan (produced from MTT by viable cells) staining reflective of the cell number and presented as means + SEM. **p*<0.05, ***p*<0.01 indicate significant difference between cell survival rate between Zyflamend and control cells (DMSO-Day 1 for pre-adipocytes and DMSO-Day 12 for mature adipocytes). †*p*<0.05, ††*p*<0.01 indicate significant difference between Zyflamend-treated mature and pre-adipocytes. #*p*<0.05, ##*p*<0.01 indicate significant difference between cells treated with the inhibitor alone and cells co-treated with Zyflamend and the indicated inhibitor. **B**) 3T3-MBX pre-adipocytes were differentiated in the presence of JNK, PKA, or apoptosis inhibitors. When indicated, a freshly prepared solution of DMSO or Zyflamend (200 μg/ml) were added at each change of media until termination of the experiments. Cells were fixed and stained with Oil Red O on days 1 and 12, then the dye was extracted and its absorbance (520 nm) quantitated. Graph represents data from three independent experiments, and are expressed as mean + SEM. **p*<0.05, ***p*<0.01 indicate significant difference between the indicated time and control cells on day one of differentiation (DMSO-Day 1). †*p*<0.05, ††*p*<0.01 indicate significant difference between Zyflamend and control (DMSO-treated) cells. #*p*<0.05, ##*p*<0.01 indicate significant difference between cells treated with the inhibitor alone and cells co-treated with Zyflamend and the indicated inhibitor. **C**) 3T3-MBX pre-adipocytes were treated with DMSO or 200 μg/ml of Zyflamend and differentiated as described in the methods section for 8 days. Cells were then lysed and immunoblotted for pHSL^S660^, HSL, cleaved caspase 3 (C-Casp.3), and ^β^-actin as a loading control. Representative immunoblots from three independent experiments are shown.

**Supplementary Table S1. Composition of Zyflamend^9^**

| **Extract** | **Latin Name** | **Amount (%)** |
| --- | --- | --- |
| Rosemary | Rosmarinus officinalis | 19.2 |
| Turmeric | Curcuma longa | 14.1 |
| Ginger | Zingiber officinale | 12.8 |
| Holy Basil | Ocimum sanctum | 12.8 |
| Organic Green Tea | Camellia sinensis | 12.8 |
| Hu Zhang | Polygonum cuspidatum | 10.2 |
| Chinese Goldthread | Coptis chinensis | 5.1 |
| Barberry | Berberis vulgaris | 5.1 |
| Organic Oregano | Origanum vulgare | 5.1 |
| Chinese Skullcap | Scutellaria baicalensis | 2.5 |
