# Supplementary figures and images for: Zyflamend, a unique herbal blend, induces cell death and inhibits adipogenesis through the coordinated regulation of PKA and JNK
Source: Adipocyte. 2020 Aug 11;9(1):454–71. doi: 10.1080/21623945.2020.1803642 (PMC7469463; doi:10.1080/21623945.2020.1803642)

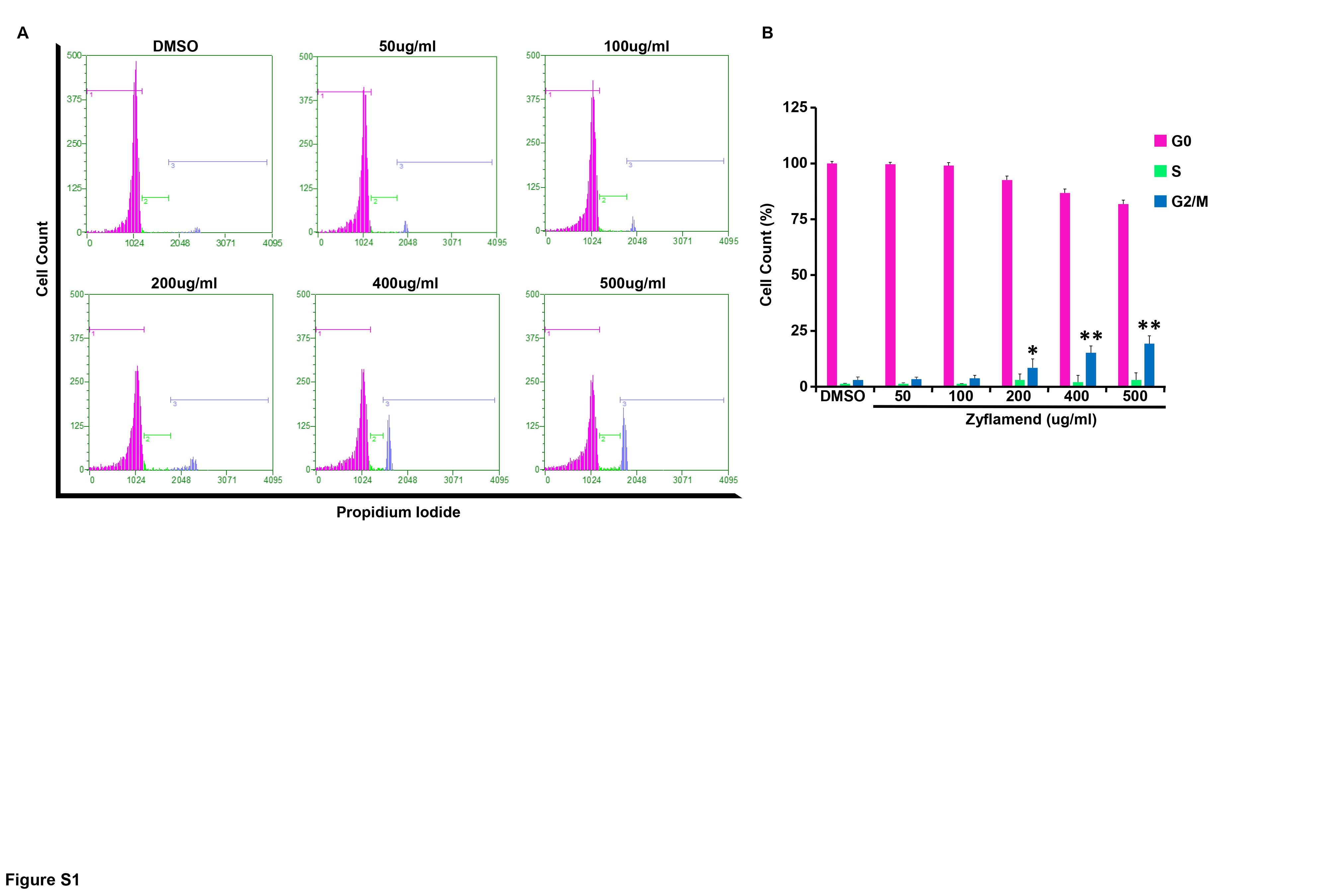

Supplement: Supplemental Material [file KADI_A_1803642_SM0903.zip › Figure S1_1.jpg]

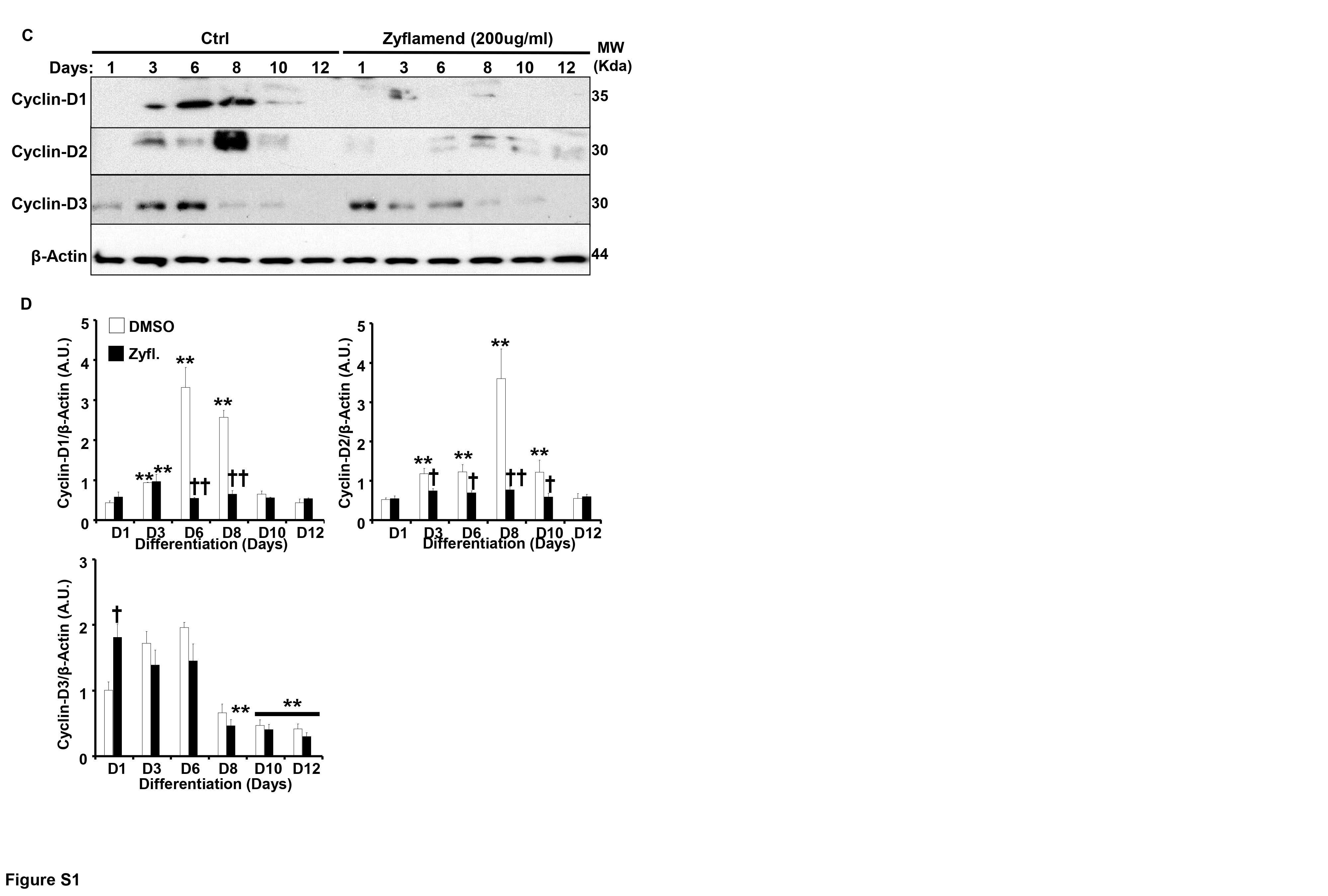

Supplement: Supplemental Material [file KADI_A_1803642_SM0903.zip › Figure S1_2.jpg]

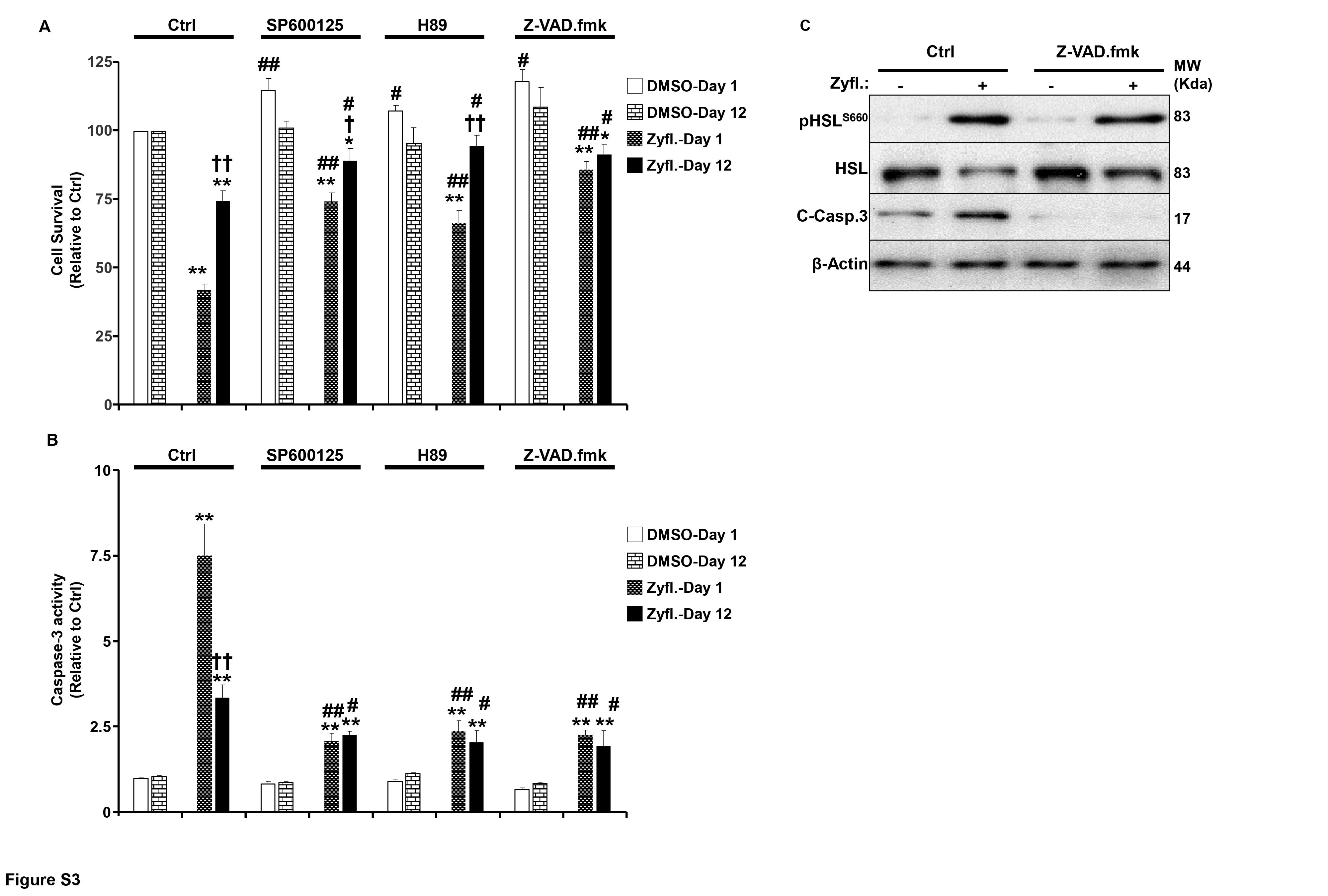

Supplement: Supplemental Material [file KADI_A_1803642_SM0903.zip › Figure S3.jpg]

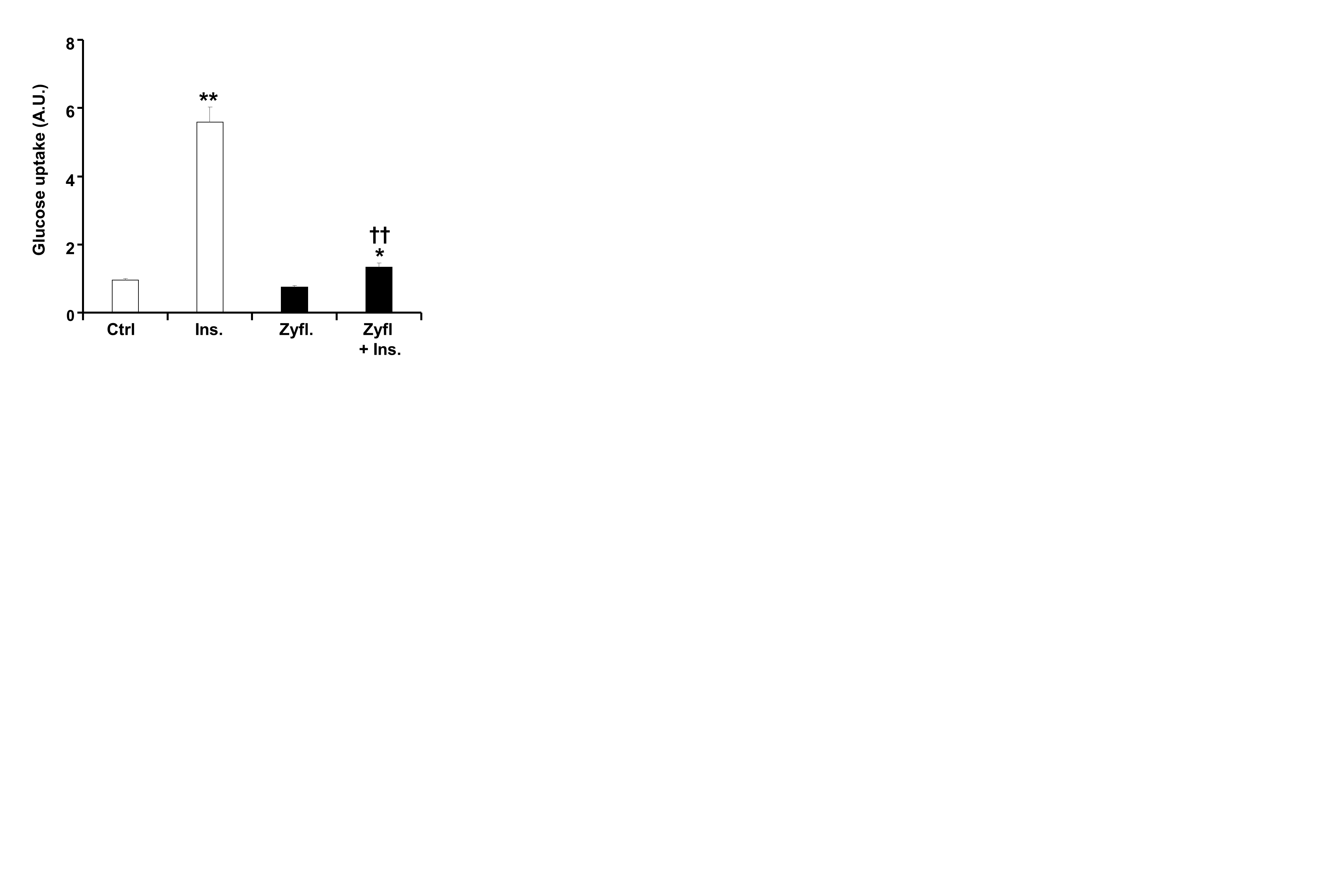

Supplement: Supplemental Material [file KADI_A_1803642_SM0903.zip › Supplementary_FigureS2.tif]
